# Retinal Ganglion Cell Atrophy in Homonymous Hemianopia due to Acquired Occipital Lesions Observed Using Cirrus High-Definition-OCT

**DOI:** 10.1155/2016/2394957

**Published:** 2016-05-04

**Authors:** Tsutomu Yamashita, Atsushi Miki, Katsutoshi Goto, Syunsuke Araki, Go Takizawa, Yoshiaki Ieki, Junichi Kiryu, Akio Tabuchi, Yasuyuki Iguchi, Kazumi Kimura, Yoshiki Yagita

**Affiliations:** ^1^Department of Sensory Science, Faculty of Health Science and Technology, Kawasaki University of Medical Welfare, Kurashiki, Okayama 701-0193, Japan; ^2^Department of Ophthalmology, Kawasaki Medical School, Kurashiki, Okayama 701-0192, Japan; ^3^Department of Neurology, The Jikei University School of Medicine, Minato-ku, Tokyo 105-8471, Japan; ^4^Department of Neurological Science, Graduate School of Medicine, Nippon Medical School, Bunkyo-ku, Tokyo 113-8603, Japan; ^5^Department of Stroke Medicine, Kawasaki Medical School, Kurashiki, Okayama 701-0192, Japan

## Abstract

*Purpose*. To report a reduction in macular ganglion cell layer and inner plexiform layer (GCL+IPL) thickness and circumpapillary retinal nerve fiber layer (cpRNFL) thickness using spectral-domain optical coherence tomography in patients with homonymous hemianopia due to posterior cerebral artery (PCA) stroke.* Methods*. Seven patients with PCA stroke were examined using Cirrus high-definition-OCT. The GCL+IPL thicknesses were divided into the hemianopic and unaffected sides. The relationship between the time after stroke and the GCL+IPL thicknesses in the hemianopic side was evaluated.* Results*. The average thicknesses of the GCL+IPL were 64.6 and 82.0 *μ*m on the hemianopic and unaffected sides, respectively, and the measurement was significantly thinner on the former side (*p* = 0.018). A regression analysis revealed a negative linear relationship (*R* ^2^ = 0.574, *p* = 0.049) between the time after stoke and the GCL+IPL thicknesses on the hemianopic side. The supratemporal and inferotemporal cpRNFL thicknesses in the eyes ipsilateral to the stroke showed a significant reduction.* Conclusion*. Our findings confirmed our previous observations that the degeneration of retinal ganglion cells can occur after PCA stroke. GCL+IPL thinning was demonstrated in the hemiretinae corresponding to the affected hemifields. Also, it is suggested that the retinal changes observed are progressive.

## 1. Introduction

Atrophy of the ipsilateral lateral geniculate nucleus (LGN) and retinal ganglion cell (RGC) in both eyes after occipital lobectomy has been reported in monkeys [1, 2]. Such atrophy is considered to be mostly due to retrograde degeneration and retinal one, transsynaptic retrograde degeneration. In humans, decreased activation of the LGN has been reported in patients with retrogeniculate hemianopia evaluated using functional magnetic resonance imaging (MRI) [3]. In the human eye, degenerated axon of the RGC, which is considered to be histological evidence of transneuronal retrograde degeneration, was reported in a patient with congenital malformations after surgical lobectomy to remove a brain tumor [4]. On the other hand, it has been suggested that transsynaptic retrograde degeneration of the RGC may not occur in adult humans based on clinical experience indicating that no optic atrophy was detected even in a patient with longstanding homonymous hemianopia [5]. However, optical coherence tomography (OCT) studies of human occipital lobe lesions have indicated that there was thinning of the circumpapillary retinal nerve fiber layer (cpRNFL), consistent with the visual field loss, using time-domain OCT (TD-OCT) [6, 7]. Jindahra et al. have reported that the mean cpRNFL thickness was significantly greater in controls than in retrogeniculate hemianopia groups using TD-OCT [6, 7].

The resolution of TD-OCT can be good, particularly for stationary subjects, but one important limitation is its slow scan speed. Spectral-domain OCT (SD-OCT) is capable of operating nearly 100 times faster than TD-OCT, making it possible to build instruments that had high scan densities with a greatly decreased time needed for the scan. Mwanza et al. [8, 9] demonstrated that the Cirrus high-definition- (HD-) OCT ganglion cell analysis (GCA) algorithm (Carl Zeiss Meditec, Dublin, CA) can successfully measure the thickness of the macular ganglion cell and inner plexiform layers (GCL+IPL) with excellent intervisit reproducibility, within a 14.13 mm^2^ elliptical annulus area centered on the fovea. Additionally, they showed that the ability of the macular GCL+IPL parameters to discriminate normal eyes from eyes with early glaucoma was comparable to that of the best cpRNFL and ONH parameters.

Using SD-OCT would make it possible to investigate whether or not there is partial optic atrophy. We recently reported the macular ganglion cell complex (GCC) thicknesses determined using RTVue-100 SD-OCT in three patients with homonymous hemianopia following unilateral posterior cerebral artery (PCA) infarction [10]. In these patients, GCC thinning was observed in accordance with the hemianopic visual field defects, despite a normal appearance of the fundus.

In this report, we used another SD-OCT instrument, Cirrus HD-OCT, to evaluate the macular GCL+IPL thickness and cpRNFL thickness in patients with homonymous hemianopia due to retrogeniculate lesions. The relationship between the time after the onset and retinal thicknesses was also investigated in a cross-sectional manner, in order to clarify whether or not the retinal changes observed were progressive.

## 2. Patients and Methods

### 2.1. Subjects

All investigations adhered to the tenets of the Declaration of Helsinki, and this study was approved by the institutional review board and the ethics committee of Kawasaki Medical School. After the nature and possible consequences of the study were explained, written informed consent was obtained from all of the participating patients.

Seven patients with acquired occipital lobe lesion were recruited. None of the patients had any history of birth trauma, headache, or head injury. Each participant underwent a comprehensive ophthalmological assessment, including the measurement of the best-corrected visual acuity, slit-lamp biomicroscopy, tonometry, dilated stereoscopic examination of the optic nerve head and fundus, color disc photography, and red-free RNFL photography. Visual fields were obtained by static automated perimetry (Humphrey Visual Field Analyzer; Carl Zeiss Meditec, Inc., Dublin, CA, USA) and/or Goldmann perimetry.

The participants included in this study met the following criteria: best-corrected visual acuity of >20/20, with a spherical equivalent between −6.0 and +3.0 diopters and a cylinder correction within ±3.0 diopters. The ocular motility, intraocular pressure, anterior segments, media, and fundus (including red-free fundus photographs) were normal in both eyes. The patients were excluded if they met any of the following criteria: a history of retinal disease, including diabetic or hypertensive retinopathy; a history of eye trauma or surgery, with the exception of uncomplicated cataract surgery; optic nerve disease including glaucoma; or a history of systemic or neurological disease other than cerebral stroke that may affect the visual field. The exclusion criteria also included evidence of more than one occipital lesion affecting visual pathways.

The eye on the same side as the stroke, which may show nasal-side hemianopia or quadrantanopia, corresponded to the uncrossed fibers at the optic chiasm (referred to as the* ipsilateral eye*). The eye on the contralateral side of the stroke, which may show temporal-side hemianopia or quadrantanopia, corresponded to the crossed fibers at the optic chiasm (referred to as the* contralateral eye*). Eyes with no apparent VF defect were classified as ipsilateral or contralateral according to the relative location of the brain damage.

Seven patients with stroke in the PCA territory were examined. All patients (three males and four females) ranging in age from 38 to 76 years old (mean, 62.4 years old) were included in the analysis (Table 1). The duration of time between the SD-OCT measurements from the onset of the occipital lobe lesion ranged from 12 months to 7.7 years (mean, 4.5 years).

### 2.2. Optical Coherence Tomography Imaging

The SD-OCT examination was performed with Cirrus HD-OCT (software version 6.0). The acquisition rate of the Cirrus HD-OCT was 27,000 A-scans per second, and the transverse and axial resolutions were 15 and 5 *μ*m, respectively. For the GCL+IPL and RNFL measurements, the Cirrus HD-OCT instrument software was used to obtain macular (macular cube 512 × 128 protocol) and optic disc scans (Optic Disc Cube 200 × 200 protocol).

The macular cube protocol included a macula thickness analysis and GCA. The GCA is designed to measure the GCL+IPL thickness within a 14.13 mm^2^ elliptical annulus (dimensions, vertical inner and outer radius of 0.5 and 2.0 mm and horizontal inner and outer radius of 0.6 and 2.4 mm, resp.) centered on the fovea with an inner vertical radius of 0.5 mm and an outer vertical radius of 2 mm, stretched horizontally by 20%. The thickness parameters derived from GCA are the average GCL+IPL thickness across the entire elliptical annulus and the thickness at six 60° sectors of the elliptical annulus. The average and six sectoral (superotemporal, superior, superonasal, inferonasal, inferior, and inferotemporal) GCL+IPL thicknesses were measured in an elliptical annulus of the macular cube scan mode. The seventh parameter was the minimum GCL+IPL measurement determined by sampling 360 spokes of measurements extending from the center of the fovea to the edge of the ellipse in 1° intervals and selecting the spoke with the lowest average. The GCL+IPL thicknesses centered on the macula were divided vertically into hemianopic and unaffected sides. The GCL+IPL thicknesses were calculated using an average for both eyes and were compared with the hemianopic/unaffected side ratio. The relationships between the time after stroke and the GCL+IPL thicknesses on the hemianopic side and the hemianopic/unaffected side ratio of the GCL+IPL thicknesses were evaluated.

The Optic Disc Cube protocol included the optic nerve head (ONH) and RNFL analyses. The Optic Disc Cube protocol scans a 6 × 6-mm^2^ area centered on the ONH to collect 200 × 200 axial scans containing 40,000 points. The ONH parameters generated and analyzed in this study were the optic disc area, rim area, average cup-to-disc ratio (CDR), vertical CDR, and cup volume. The RNFL parameters calculated by the protocol and analyzed in this study were the average RNFL thickness (average RNFL thickness at the 3.46 mm diameter circle centered on the ONH); temporal, superior, nasal, and inferior quadrant RNFL thicknesses, and the RNFL thickness at 12 clock hours (30-degree segments of the measurement circle).

Good quality scans were defined as those with a signal strength of six or higher, without RNFL discontinuity or misalignment, involuntary saccade, or blinking artifacts and an absence of algorithm segmentation failure on a careful visual inspection.

### 2.3. Statistical Analysis

The statistical analyses were performed using the IBM SPSS Statistics version 21.0 software program (SPSS Japan, Inc., Tokyo, Japan). The data are presented as the means ± SD. Differences in the GCL+IPL thicknesses between the two sides were analyzed using a Wilcoxon signed-rank test. The relationships between the GCL+IPL on hemianopic sides, the hemianopic/unaffected side ratio of the GCL+IPL thicknesses, and the time after stroke were evaluated with linear and second-order polynomial regression analyses. A Wilcoxon signed-rank test was used to compare the average cpRNFL thicknesses between the ipsilateral eyes of patients with cerebral stroke and contralateral eyes. For all analyses, values of *p* < 0.05 were considered to be statistically significant.

## 3. Results

**Figure 1:**
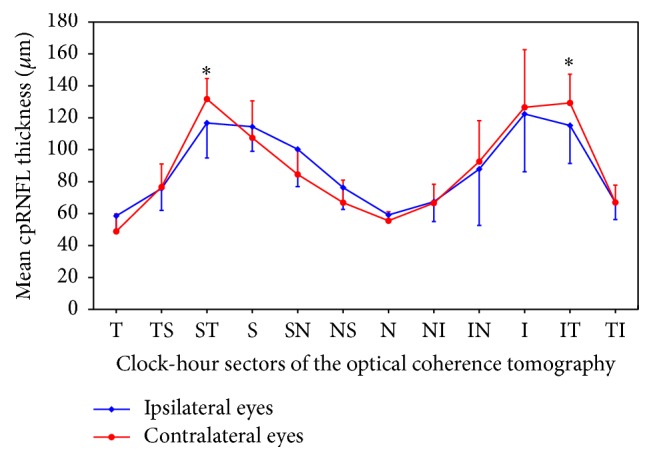
A graph showing the circumpapillary retinal nerve fiber layer (cpRNFL) thickness in the ipsilateral eyes of patients with PCA stroke compared with contralateral eyes. I, inferior; IN, inferonasal; IT, inferotemporal; N, nasal; NI, nasal-inferior; NS, nasal-superior; S, superior; ST, supratemporal; SN, supranasal; T, temporal; TI, temporal-inferior; TS, temporal-superior. ^∗^ *p* < 0.05 between groups.

The average thickness of the GCL+IPL was 64.6 ± 15.8 and 82.0 ± 7.8 *μ*m on the hemianopic and unaffected sides, respectively, and was significantly thinner on the hemianopic side (*p* = 0.018) (Table 2). The supratemporal and inferotemporal cpRNFL thicknesses in the eyes ipsilateral to the stroke showed a significant reduction (*p* < 0.05) compared with those in the fellow eyes. There was no statistically significant difference in the cpRNFL thickness between the eyes in any of the other sectors (Figure 1).

**Figure 2:**
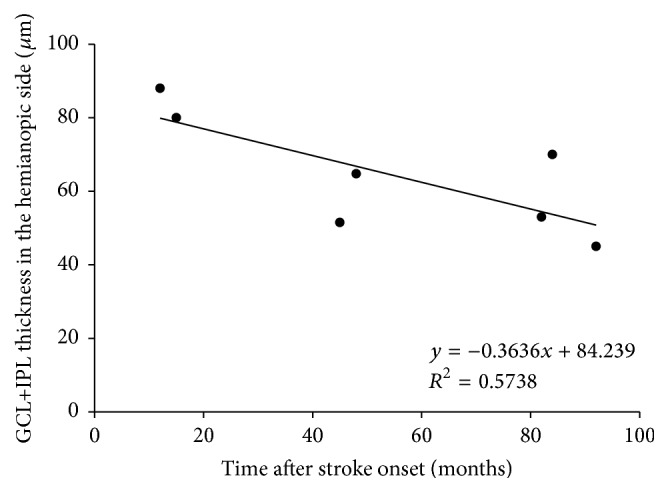
A regression analysis revealed a negative linear relationship (linear regression, *R* ^2^ = 0.574, *p* = 0.049) between the time after stroke and GCL+IPL thicknesses in hemianopic eyes.

**Figure 3:**
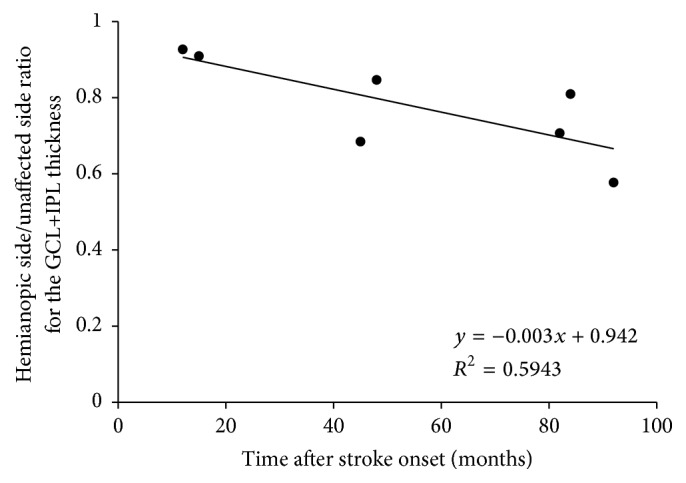
The ratio of the GCL+IPL thickness on the hemianopic side to that on the unaffected side was significantly correlated with the time after stroke (*R* ^2^ = 0.594, *p* = 0.042).

A regression analysis revealed a negative linear relationship (linear regression, *R* ^2^ = 0.574, *p* = 0.049) between the time after stroke and the GCL+IPL thicknesses in hemianopic eyes (Figure 2). Additionally, the ratio of the GCL+IPL thickness on the hemianopic side to that on the unaffected side was significantly correlated with the time after stroke (*R* ^2^ = 0.594, *p* = 0.042) (Figure 3).

## 4. Case Reports

### 4.1. Case 1

In January 2009, a 66-year-old female with diabetes mellitus suddenly noticed a left-sided visual field defect. The best-corrected visual acuity was 1.0 OU. The ocular motility, intraocular pressure, anterior segments, media, and fundus (including red-free fundus photographs) were normal in both eyes. Static automated perimetry showed complete left homonymous hemianopia with macular splitting. MRI revealed an infarction of the right PCA territory. This patient was also examined using RTVue-100 OCT and has been reported previously [10].

**Figure 4:**
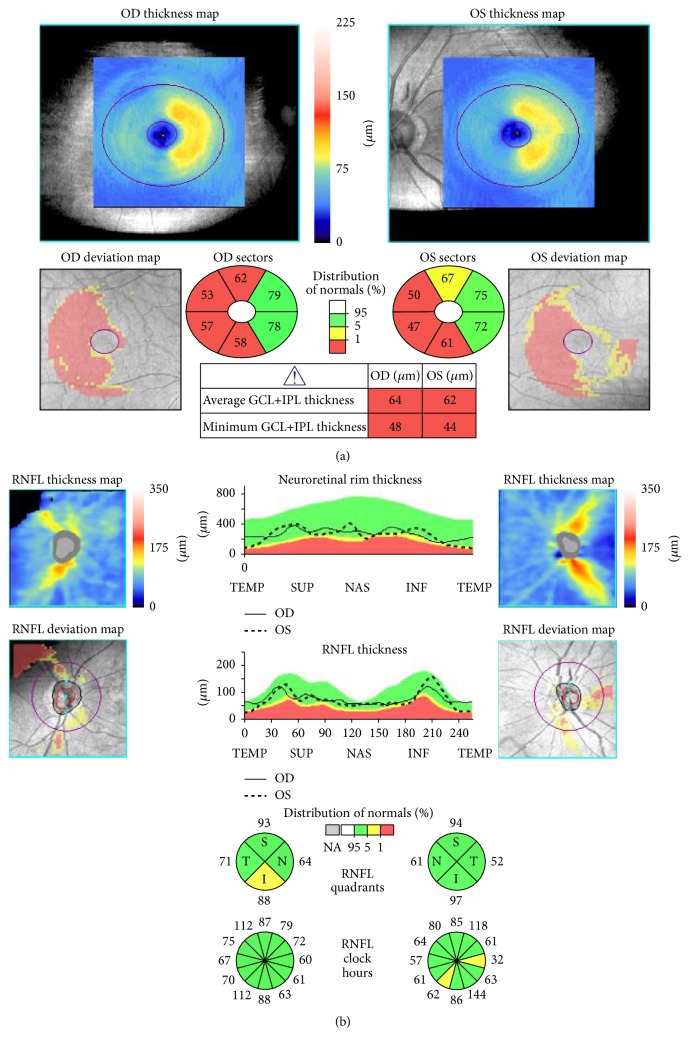
Case 1. (a) GCL+IPL thinning was observed in the temporal retina of the RE and the nasal retina of the LE. An abnormal area (yellow: outside of the 95% normal limit, red: outside of the 99% normal limit) in the deviation map was present that corresponded to the hemianopic visual field defects. (b) Cirrus HD-OCT images of the retinal nerve fiber layer (RNFL) around the optic disc. In the cpRNFL thickness map, T indicates temporal; S, superior; N, nasal; I, inferior. A, the 3- and 7-clock-hour retinal nerve fiber layer (RNFL) thicknesses were on the borderline in the RNFL clock-hour sector map for the left eye. The deviation map of the cpRNFL thickness was significantly thinner in the superior and inferior regions of the right eye and the temporal and inferior regions of the left eye compared to normative database values.

In October 2012, areas with GCL+IPL thinning in both eyes were found in accordance with the hemianopic visual field defect (temporal retina of the right eye and nasal retina of the left eye) (Figure 4(a)). In the deviation map of the cpRNFL thickness, there were areas with significant thinning in the superior and inferior portions in the right eye and nasal and inferior portions in the left eye (Figure 4(b)).

### 4.2. Case 7

**Figure 5:**
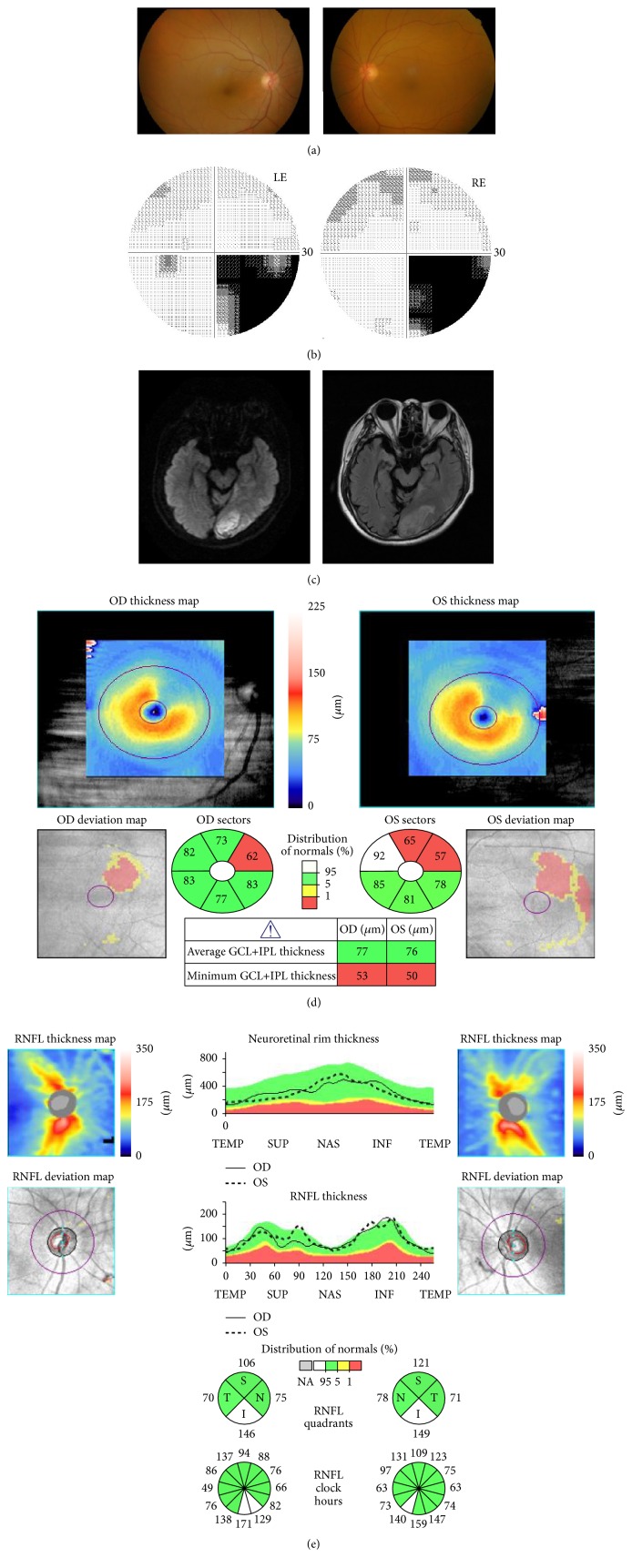
Case 7. (a) Fundus photographs at the time of OCT. (b) Visual fields obtained by static automated perimetry showing right inferior homonymous quadrantanopia. (c) Initial DWI revealed a hyperintense lesion on the left occipital lobe (left). One day after onset, a FLAIR image demonstrated a hyperintense lesion, which represented an acute phase of hemorrhagic stroke (right). (d) GCL+IPL thinning was observed in the superior nasal retina of the RE and the superior temporal retina of the LE. Similarly, an abnormal area in the deviation map was present corresponding to the hemianopic visual field defects. (e) The cpRNFL clock-hour sector map showed that all cpRNFL thicknesses in both eyes were within the normal range.

In April 2005, a 76-year-old male was found to have right-sided visual field defects. The best-corrected visual acuity was 1.0 OU. The ocular motility, intraocular pressure, anterior segments, media, and fundus were normal in both eyes (Figure 5(a)). Static automated perimetry showed right inferior homonymous quadrantanopia (Figure 5(b)). MRI revealed a cerebral hemorrhage in the left PCA territory (Figure 5(c)).

In May 2012, GCL+IPL thinning of both eyes was observed in accordance with the affected quadrants (superior nasal retina of the right eye and superior temporal retina of the left eye) (Figure 5(d)). The cpRNFL thickness OU was within the normal range in both eyes (Figure 5(e)).

## 5. Discussion

Retrograde degeneration of the RGCs may be detected by OCT in humans with cerebral damage. Previous studies examined a large number of patients with cerebral infarction in various locations [11, 12], but it was unclear whether each of the patients did have homonymous visual field defects. In the present study, we included only patients with homonymous hemianopia due to occipital lobe lesions and excluded those with hemianopia due to an optic tract lesion or unclear cause. Using Cirrus HD-OCT, we found that GCL+IPL thinning occurred in both eyes, corresponding to the homonymous visual field defects following PCA stroke.

The early OCT studies in homonymous hemianopia detected a reduction in the cpRNFL thickness [6, 7, 11, 13], which we also confirmed in the areas consistent with the visual field defects and known topographical distribution of RGCs around the optic disc. Other recent reports examined the inner macular retinal thicknesses [10, 12, 14–19], which clearly showed a hemianopic thinning pattern, that is, nasal thinning in one eye and temporal thinning in the other. By using SD-OCT (RTVue), we previously found that GCC thinning occurred in the retina corresponding to the homonymous hemianopia following PCA infarction [10]. Similarly, using a different SD-OCT instrument, Tanito and Ohira [14] reported a patient with reduced inner macular retinal thickness after an occipital lobe infarction, in whom RGC loss was seen in a wide area in the macula corresponding to the hemianopia. These findings indicate that retrograde degeneration of RGCs after cerebral infarction can be detected* in vivo* by measuring the GCL+IPL or GCC thickness using SD-OCT. Degeneration of the ipsilateral optic tract in patients with homonymous hemianopia caused by occipital lobe lesions has also been demonstrated with MRI, which was attributed to transneuronal retrograde degeneration of the RGC [20–22].

The scanning area placement of the RTVue may be a better fit for the detection of early glaucomatous damage in the macular region, which preferentially affects temporal sites in the parafoveal region. Cirrus OCT scans oval areas, centered on the fovea. Although the software used for RTVue does not separate the RNFL and GCL, Cirrus HD-OCT allows for the separation of RNFL from the GCL at the macula. It is hypothesized that Cirrus HD-OCT, which excludes the macular NFL from the GCC, would improve the structure-function relationship, because some of the nasal nerve fibers in the macular region originate from the temporal retina. Previous OCT studies from other groups using Cirrus OCT also found significant thinning of the macular GCL+IPL corresponding to the visual field defects in patients with retrogeniculate homonymous hemianopia [12, 15–19].

Although previous OCT studies demonstrated homonymous thinning of the retina supporting the concept of transneuronal retrograde degeneration, patients with lesions of the optic tract or lateral geniculate nucleus, which lead to direct retrograde degeneration, were included in some of the studies [7, 13, 16], thus complicating the interpretation of the results. Although previous OCT studies attributed the inner retinal thinning to transsynaptic degeneration [6, 7, 11, 13, 15], the evidence supporting this degeneration has not yet been unambiguously shown. However, in order to prove that the changes we observed were transsynaptic, the direct damage to RGCs needed to be excluded. Since the previous OCT studies that attributed the retinal thinning to transsynaptic degeneration of RGCs did not show MRI images or report details of the area of brain damage [6, 13, 15, 16], it is not possible to determine whether the assumption holds true. Instead, the brain lesions causing extensive inner retinal thinning are almost always close to the lateral geniculate nucleus in the MRI images shown as an example in such papers [7, 10, 11]. Therefore, although the patients with homonymous hemianopia frequently have primary lesions in the optic radiation or the visual cortex, the blood supply to the lateral geniculate nucleus may also be compromised.

The supratemporal and inferotemporal cpRNFL thicknesses in the eyes ipsilateral to the stroke were significantly decreased by the degeneration of the RGCs, where the uncrossed fibers from the damaged brain are located. A previous study showed that the thinning was more pronounced in the nasal cpRNFL of the contralateral side and in the temporal cpRNFL of the ipsilateral side to the cerebral damage [11]. Although the difference did not reach statistical significance, the cpRNFL of the nasal sectors was slightly thinner in the contralateral eyes than in the ipsilateral eyes, so the discrepancy between our results and those reported by Park et al. [11] is likely due to the differences in the sensitivity of the comparison. We performed an intrasubject comparison, while Park et al. [11] compared each eye with control subjects' eyes. These findings are generally in line with the known topographical arrangement of retinal nerve fiber layers, that is, RGC axons, but the inner macular thinning is easier to interpret, because the change respects the vertical meridian.

The GCL+IPL thickness on the hemianopic side was significantly correlated with the time after stroke and was thinner in patients with older brain damage (Figure 2). This cannot be explained simply by physiological aging, because the ratio of the hemianopic side to the unaffected side of the GCL+IPL showed a similar trend (Figure 3). cpRNFL thinning has been shown to be progressive in the first years after the cerebral damage, subsequently becoming relatively stable or progressing more slowly in later years [7]. Park et al. [11] also reported that the RNF thickness was significantly related to the time after stroke onset and the location of the infarction. The relationship found in OCT studies is similar to that calculated from RGC counts in experimental occipital lobe lesions in monkeys [2]. Although the follow-up periods were relatively short, longitudinal OCT studies revealed progressive thinning of the cpRNFL corresponding to the hemianopic visual field defects [7, 23].

Our findings confirmed our previous observation that the degeneration of RGCs can occur after PCA stroke. GCL+IPL thinning was demonstrated in the hemiretinae corresponding to the affected hemifields. The supratemporal and inferotemporal cpRNFL thicknesses in the eyes ipsilateral to the stroke were significantly decreased by the degeneration of the RGCs, where the uncrossed fibers from the damaged brain are located. Our results suggest that the reduction in the GCL+IPL thickness is slowly progressive over a period of several years after the onset of PCA stroke. We performed a cross-sectional analysis in this study to estimate the time course of the changes in retinal thickness, but ideally, a longitudinal analysis in the same patients for many years is required to fully understand the time course.

## Figures and Tables

**Table 1:** The patient demographics.

Case	Age	Gender	Cause	Visual field
1	69	Female	Posterior cerebral artery infarction	LHH
2	52	Female	Posterior cerebral artery infarction	LHH
3	66	Male	Posterior cerebral artery infarction	LHH
4	73	Male	Posterior cerebral artery infarction	LHH
5	63	Female	Posterior cerebral artery infarction	RHH
6	38	Female	Posterior cerebral artery infarction	RHH
7	76	Male	Posterior cerebral artery hemorrhage	RIQ

HH, homonymous hemianopia; IQ, inferior quadrantanopia.

**Table 2:** The macular ganglion cell layer and inner plexiform layer thicknesses in patients with homonymous hemianopia and the time after stroke.

Case	GCL+IPL (*μ*m)		Hemianopic side/unaffected side ratio	Months after stroke
	Hemianopic side	Unaffected side		
1	51.5	75.3	0.68	45
2	53.0	75.0	0.71	82
3	80.0	88.0	0.91	15
4	64.8	76.5	0.85	48
5	45.0	78.0	0.58	92
6	88.0	95.0	0.93	12
7	70.0	86.5	0.81	84
Avg	64.6	82.0	0.78	54.0
